# HMGB1 and mitochondrial dysfunction: a double-edged sword in ageing

**DOI:** 10.1098/rsob.240376

**Published:** 2025-10-15

**Authors:** Maheen Wahid, Margaret Cunningham

**Affiliations:** ^1^ Strathclyde Institute of Pharmacy and Biomedical Sciences, Glasgow, UK

**Keywords:** HMGB1, ageing, mitochondrial dysfunction

## Abstract

Ageing is an intricate and progressive decline across all biological systems, marked by various molecular and cellular processes termed as the hallmarks of ageing. One of the hallmarks is mitochondrial dysfunction, which is brought about through several pathways: mutations in mitochondrial DNA, elevated reactive oxygen species production, disrupted mitochondrial dynamics and impaired mitophagy. Here, we explore the role of high mobility group box 1 (HMGB1) as a potential contributor to mitochondrial dysfunction, examining how it may influence these pathways through its dual roles as both a protector of mitochondrial integrity and a promoter of inflammatory damage. Furthermore, we consider how mitochondrial dysfunction, possibly mediated by HMGB1, could link to other hallmarks of ageing, positioning HMGB1 as a possible central regulator in the ageing process.

**Figure:**
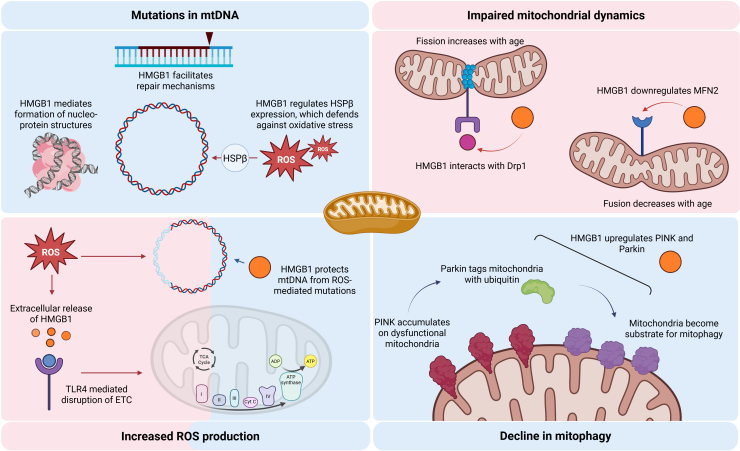

## 1. General introduction

Ageing is a complex and multifactorial biological process involving a progressive decline of physiological, molecular and biochemical processes in the body [1]. Gaining an apt understanding of the intricacy of ageing in humans is imperative, owing to prolonged life expectancy as well as the increasing proportion of the geriatric population globally [2]. It is estimated that the percentage of individuals living beyond the age of 60 years will increase from 12% to about 22% by the end of 2050 [2]. Almost 13 million people in the United Kingdom (UK) are older than 65 [3]. This increase in lifespan is one of the biggest feats in the last century; however, it must be recognized that life expectancy has increased across all age groups [4] (e.g. in childhood for reduction of infectious diseases [5]). While the extension of lifespan is a significant achievement, the number of years an individual lives in good health, free from serious illness or disability (i.e. the healthspan) is now a target towards which endeavours in research are directed [6]. This focus is crucial as the rise in the ageing population places a growing burden on healthcare systems globally [7–9]. In recent years, both long-established organizations such as the National Institute on Aging (NIA) (founded in 1974) and newer initiatives including the UK Ageing Network (UKAN) and Targeting Ageing with Metformin (TAME) have increasingly focused on extending healthspan and addressing chronic illnesses of old age [6,10].

**Figure 1.**
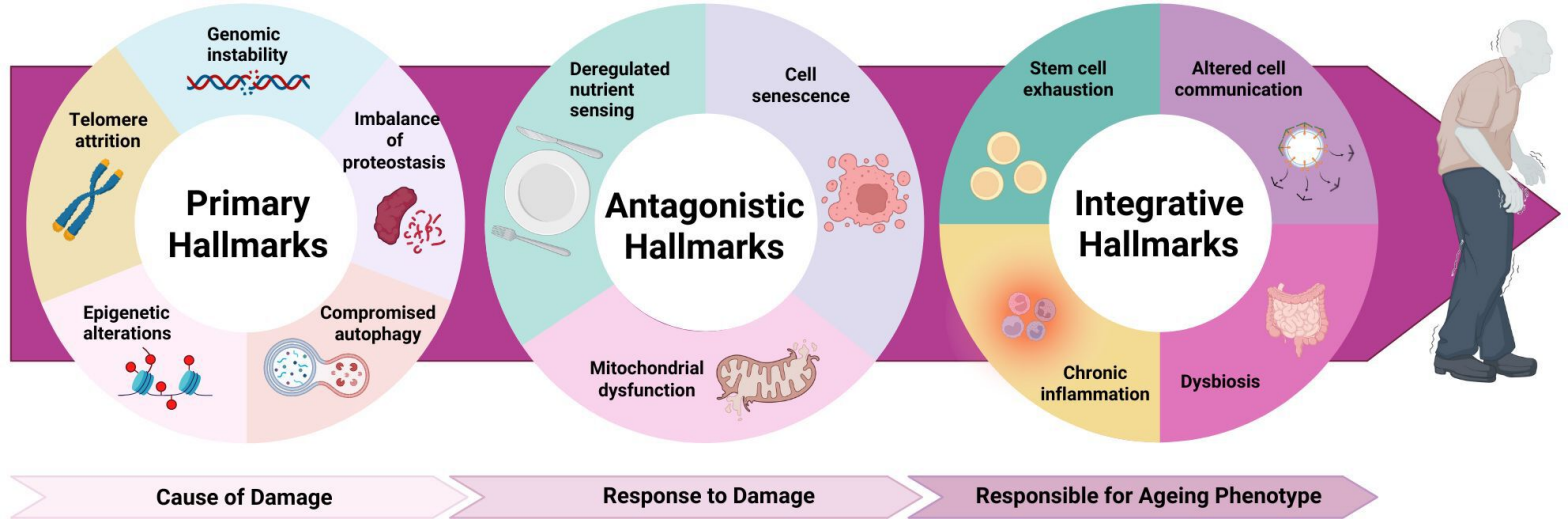
The hallmarks of ageing, sorted into primary, antagonistic and integrative categories. The figure shows 12 hallmarks of ageing, all of which cumulatively contribute to physiological decline seen with increasing age. Figure created using BioRender.

As the pursuit of extension of healthspan gains momentum, researchers in the field of geroscience have identified 12 key processes, termed as the hallmarks of ageing, that essentially drive the ageing process [11]. They are categorized into primary, antagonistic and integrative hallmarks (figure 1). The three hallmark categories do not work autonomously but are intricately connected in a hierarchical manner—the primary hallmarks trigger the ageing process, and the injury produced by them builds up with age. The antagonistic hallmarks, which offer cellular benefits initially, become harmful with time, partly owing to the cell injury brought about by the primary hallmarks. Lastly, the damage manifested by the former two hallmarks accumulates and saturates the body ability to compensate—integrative hallmarks of ageing appear as a physiological consequence. Hence, these interconnected processes contribute to the progressive functional decline seen in ageing and age-related diseases.

The hallmarks of ageing provide a valuable conceptual framework for understanding and addressing the biological processes that underlie health and longevity. By categorizing these processes, researchers can target specific mechanisms and pathways, facilitating the development of interventions aimed at improving healthspan. This framework not only clarifies the physiological drivers of ageing but also guides research efforts across disciplines, focusing on preserving function and preventing age-related decline.

One antagonistic hallmark, mitochondrial dysfunction, is strongly implicated in the ageing process and contributes to various ageing phenotypes and age-related disorders through increased production of reactive oxygen species (ROS), oxidative stress and disruption of cellular homeostasis [12]. While mitochondrial dysfunction was initially considered the primary driver of ageing [13], recent multi-omics research suggests a more complex nonlinear pattern of molecular changes, beyond the mitochondria at the system level, that take place across distinct periods of the lifespan [14].

The accumulation of dysfunctional mitochondria is closely associated with chronic inflammation, which can exacerbate the detrimental effects of oxidative stress and further compromise cellular integrity. The ROS generated from dysfunctional mitochondria cause permeabilization of the mitochondrial membrane. When ROS and mitochondrial DNA (mtDNA) leak out into the cytosol, this results in inflammation and ultimately, cell death [15]. Hence, a highly permeable mitochondria can act as a latent stimulus for inflammation, which in itself, is an ageing hallmark. This interplay between mitochondrial dysfunction and inflammation highlights the potential for certain molecules to act as mediators that bridge these two processes. One such molecule is high mobility group box 1 (HMGB1), a damage-associated molecular pattern (DAMP) that not only plays a crucial role in inflammatory responses but may also be intricately linked to mitochondrial health. Interestingly, mitochondrial DAMP (mtDAMP) signalling pathways have been implicated as regulators that elicit mitochondrial-dependent inflammation [16,17]. HMGB1 has not yet been implicated in mtDAMP signalling; however, this is an emerging area that is still in its infancy.

HMGB1 is a highly conserved, non-histone chromatin-binding protein that plays a pivotal role in maintaining cellular homeostasis. Under physiological conditions, it resides predominantly in the nucleus, where it binds to both DNA and histones to stabilize nucleosome structure and shield genetic material from damage [18]. By modulating histone–DNA interactions, HMGB1 influences chromatin compaction and enables processes such as nucleosome sliding that facilitate transcription and DNA repair [19,20]. While traditionally known for its nuclear role, HMGB1 also functions as a pro-inflammatory mediator when released into the extracellular space following cellular stress or injury. Once released, HMGB1 interacts with receptors such as toll-like receptor 4 (TLR-4) and the receptor for advanced glycation end products (RAGE), mediating inflammation and ultimate cellular decline [21].

Beyond its role in inflammation, HMGB1 is increasingly recognized as a regulator that affects multiple hallmarks of ageing, including chronic inflammation, cellular senescence and disrupted autophagy [22]. Reduced intracellular levels of HMGB1 compromise cellular homeostasis through multiple mechanisms: in fibroblasts, HMGB1 deficiency has been linked to loss of telomere stability and impaired telomerase activity [23], while in neurons, age-related decline of nuclear HMGB1 has been associated with increased DNA damage [24]. Conversely, excessive extracellular release of HMGB1 promotes maladaptive inflammation and tissue damage, as shown in senescent fibroblasts where HMGB1 secretion sustains pro-inflammatory cytokine release [25], and in the ageing brain where increased extracellular HMGB1 contributes to neuroinflammatory priming and cognitive decline [26]. These age-associated alterations position HMGB1 as both a potential biomarker and a therapeutic target aimed at promoting healthy ageing.

Emerging evidence suggests that HMGB1 plays a significant role in mitophagy and mitochondrial quality control [27]. However, mitochondrial dysfunction is a complex process influenced by various factors. While functional studies linking HMGB1 to age-associated mitochondrial dysfunction are currently lacking, a thorough review of the literature may reveal potential connections between HMGB1 and the underlying mechanisms contributing to mitochondrial impairment. This exploration could enhance our understanding of role of HMGB1 in the broader context of age-related mitochondrial dysfunction. Moreover, since mitochondrial dysfunction is closely linked with other hallmarks of ageing [12], examining HMGB1 in this context could reveal new connections between these hallmarks and help identify potential therapeutic targets for mitigating ageing-related damage.

## 2. Purpose of the review

This review will examine the influence of HMGB1 on mitochondrial dysfunction within the ageing process, with a focus on how it may act as a central regulator linking various hallmarks. Uncovering these connections could offer new insights into therapeutic strategies that target mitochondrial health to improve healthspan.

### 2.1. Age-associated mitochondrial dysfunction: drivers and HMGB1 interactions

Mitochondrial dysfunction during ageing is driven by several interconnected factors, notably mutations in mtDNA, increased production of ROS, impaired mitochondrial dynamics and dysregulated mitophagy [12]. HMGB1 emerges as a complex regulator within this context, potentially stabilizing mtDNA through repair mechanisms while also interacting with processes linked to oxidative stress. In this section, we expand upon causative mechanisms behind mitochondrial function impairment, and potential link to HMGB1.

### 2.2. Mutations in mtDNA

Unlike nuclear DNA, mtDNA is particularly vulnerable to genomic instability, showing a susceptibility that is up to 15-fold higher with age [28]. This vulnerability arises from several unique factors. The high replication rate of mitochondria leads to a higher probability of replication errors and, consequently, mutations in mtDNA over time [29]. Moreover, mitochondria operate in a ROS-rich environment, exposing mtDNA to oxidative stress that contributes to DNA damage and mutation. Lastly, unlike nuclear DNA, mtDNA lacks histones, which provide a protective cover for nuclear DNA, leaving the compact mtDNA more exposed to potential damage [30]. With ageing, both point mutations and deletions in mtDNA increase [31,32]. These accumulated mutations have been linked to a shorter lifespan in murine ageing models, highlighting the impact of mtDNA stability on longevity [33].

The accumulation of mtDNA mutations over time highlights the importance of robust repair mechanisms in preserving mitochondrial health. HMGB1 plays a pivotal role in maintaining this stability. As a chromatin-associated protein, HMGB1 may extend its regulatory influence to mitochondria, assisting in the repair of damaged mtDNA, effectively reducing mutagenesis and promoting genomic stability. HMGB1 is present in the mitochondrial membrane and binds to, as well as repairs, damaged mtDNA in a murine model of spinocerebellar ataxia [34]. In this study, inhibition of HMGB1 increased mtDNA damage and potentiated neurodegeneration of Purkinje cells. Repair of mtDNA can follow multiple routes, with base excision repair (BER) being the primary pathway. DNA bases that have undergone oxidation, deamination, methylation or alkylation are removed and repaired via BER [35]. HMGB1 has been identified as a cofactor for BER, with potential regulatory influence on key enzymes in this pathway, which include apurinic/apyrimidinic endonuclease, DNA polymerase β, and FEN1 and Flap endonucleases [36].

Mismatch repair (MMR) and double-strand break (DSB) repair pathways have also been shown to be present in the mitochondria [37,38], and role of HMGB1 in excision of damaged DNA base pairs following these pathways has been documented [39,40].

HMGB1 physically interacts with MutSα, a DNA MMR protein that identifies the DNA base pairs that have been incorrectly incorporated [39], and stimulates exonuclease excision [41]. It has also been implicated in the variable–diversity–joining rearrangement (V(D)J) recombination sub-pathway of DSB repair, where HMGB1 and HMGB2 enhance RAG-mediated cleavage of recombination signal sequences, improving adherence to the 12/23 rule crucial for accurate recombination [40]. However, direct genetic evidence confirming an essential role for HMGB1 in V(D)J recombination *in vivo* is still lacking.

Beyond direct repair, HMGB1 also regulates mitochondrial bioenergetics, as HMGB1 knockdown has been shown to lead to a decline in mitochondrial oxidative phosphorylation as well as altered mitochondrial morphology [27]. These effects are mediated via regulation of heat shock protein beta-1 (HSPβ1) expression by HMGB1 and serve as defence against mitochondrial dysfunction under stress.

Interestingly, mtDNA can condense into nucleo-protein structures termed as mitochondrial nucleoids. These compact structures act as shields against damage to mtDNA [42], and HMG group of proteins mediate formation of these structures, reinforcing role of the protein in providing protection to mtDNA from genomic instability [43]. Collectively, these mechanisms emphasize HMGB1 multifaceted role in preserving mtDNA integrity—from its direct involvement in DNA repair pathways to its regulatory effects on mitochondrial bioenergetics and nucleoid stability.

### 2.3. Increased ROS production

Ageing is also associated with a rise in ROS production within mitochondria, particularly at Complex I and Complex III of the electron transport chain (ETC) [44]. The enzymatic activity of these complexes is compromised with age, leading to increased ROS levels, which, in turn, cause breaks and oxidative modifications in the mtDNA. This damage can introduce mutations in genes encoding ETC components and ATP synthase, further impairing mitochondrial function. The resulting dysfunction generates a loop, where increased ROS production exacerbates mtDNA damage, perpetuating mitochondrial decline with age [45].

While the direct damage caused by ROS to mtDNA is well-documented [46,47], the involvement of HMGB1 in this context adds another layer of complexity. On the one hand, HMGB1 is instrumental in mtDNA repair and maintenance, helping to stabilize mtDNA and mitigate genetic instability. However, under conditions of elevated ROS and oxidative stress, HMGB1 translocates from the nucleus to the cytoplasm and is released into the extracellular space, where it serves as an inflammatory mediator causing cell damage via TLR-4-mediated downstream signalling [48]. Activation of TLR−4 has also been implicated in disrupted ETC complex function and altered mitochondrial enzyme activity [49]. In case of ROS-induced release, therefore, HMGB1 shifts from its DNA-protective role to a promoter of mitochondrial dysfunction and cellular injury. Moreover, the extracellular release of HMGB1 positions it as a DAMP, extending its involvement to the TLR-9 signalling pathway. During hypoxic conditions, HMGB1 can bind to mtDNA released from damaged mitochondria, forming a complex that activates TLR-9. This pathway has been linked to tumour cell proliferation, and highlights the potential role of HMGB1 as a mtDAMP [50].

Furthermore, the inhibition of HMGB1 has been shown to alleviate oxidative stress-induced mitochondrial dysfunction by reducing ROS levels and mitochondrial fragmentation, as well as restoring mitochondrial membrane potential [51]. These findings highlight its role in perpetuating the oxidative stress cycle. Hence, HMGB1 plays a dual role in mitochondrial function, acting as both a protector and a potential antagonist.

One emerging explanation for these paradoxical effects lies in the oxidation state of HMGB1 [52]. The protein can exist in distinct redox isoforms: the fully reduced form, which promotes chemotaxis and tissue repair, and the disulfide form, which activates pro-inflammatory signalling through TLR-4. Under conditions of oxidative stress, HMGB1 undergoes thiol oxidation to form the disulfide isoform, shifting its role from protective to inflammatory [53]. In parallel, other post-translational modifications (PTMs) influence HMGB1 function by controlling its localization and secretion. Acetylation drives HMGB1 translocation from the nucleus to the cytoplasm in activated monocytes, redirecting it towards secretion and enabling its extracellular signalling role [54], while phosphorylation by protein kinase C reduces DNA-binding affinity and nuclear retention, further biasing HMGB1 towards secretion [55]. These combined mechanisms illustrate how redox-driven functional switching, together with PTM-mediated regulation of localization, allows HMGB1 to exert context-dependent effects. This dualism is consistently observed across tissues. In the gastrointestinal barrier, cytosolic HMGB1 safeguards autophagy while restraining apoptosis during inflammation [56]; in skeletal muscle, it contributes to regeneration under physiological conditions but exacerbates pathology in muscular dystrophy [57]. These post-translational control mechanisms provide a biochemical framework through which cellular stressors such as ROS may shape context-dependent effects of HMGB1.

### 2.4. Impaired mitochondrial dynamics

As mitochondria age, disruptions in critical processes such as fusion, fission and biogenesis compromise their functional integrity [58]. Mitochondrial fusion facilitates the exchange of mitochondrial matrix components, thereby mitigating localized damage and maintaining bioenergetic efficiency. Mitochondrial fusion is mediated by several proteins, including mitofusins. Mitofusin 2 (MFN2) plays a crucial role in mitochondrial function and ageing. Research shows that MFN2 expression decreases with age in skeletal muscle, contributing to mitochondrial dysfunction and sarcopenia [59]. This decrease may impair autophagy and mitochondrial quality control, leading to accumulation of damaged mitochondria [59,60]. Alternatively, overexpression of MFN2 in murine models reversed the impairment in autophagy, mitigating age-related decline in skeletal muscle mass and function [61]. In oocytes, MFN2 deletion results in mitochondrial dysfunction, shortened telomeres, and accelerated follicular depletion, mimicking reproductive ageing [62]. These alterations in mitochondrial dynamics may be influenced by HMGB1. Elevated levels of HMGB1 have been shown to downregulate MFN2 expression in CD4+T lymphocytes, potentially disrupting mitochondrial dynamics and contributing to immune dysfunction [63]. This reduction in MFN2 disrupts mitochondrial dynamics and activates the mitochondrial apoptotic pathway through elevated intracellular calcium concentration and caspase activation [64]. This connection underscores the interplay between mitochondrial health and the inflammatory processes mediated by HMGB1 [59,60].

Mitochondrial fission is crucial for the segregation of damaged mitochondrial fragments, enabling their selective removal via mitophagy [65]. With increasing age, the mitochondrial dynamics shift from fusion to fission, with an increase in fragmented mitochondrial structures within the cell [66]. In mammals, dynamin-related protein 1 (Drp1), present on the outer mitochondrial protein, is considered the master regulator of mitochondrial fission [67]. Both knockdown and overexpression of Drp1 lead to dysfunctional mitochondria. Under ischaemic conditions, immunoelectron microscopy revealed HMGB1 to be expressed in the mitochondria and peroxisomes, where it was found to be colocalized with Drp1 [68], with results suggesting a functional role for HMGB1 in mitochondrial fission. Interestingly, HMGB1 increases Drp1 phosphorylation, leading to mitochondrial fragmentation, with Drp1 knock down preventing HMGB1-dependent mitochondrial fission [69].

### 2.5. Decline in autophagy and mitophagy

The decline in autophagy and mitophagy is a hallmark of ageing that jeopardizes mitochondrial quality control. Mitophagy is a form of selective autophagy process that eliminates damaged mitochondria to maintain cellular health. Defective mitophagy leads to the accumulation of damaged mitochondria and cellular dysfunction [70]. Interestingly, pharmacological induction of mitophagy with urolithin A has been shown to attenuate age-associated inflammation and improve neurological function in old mice [71], suggesting that targeting mitophagy could be a promising therapeutic strategy for managing age-related diseases and promoting healthspan [72].

The PTEN-induced putative kinase-1 (PINK1)/Parkin pathway plays a crucial role in mammalian mitophagy by selectively targeting damaged mitochondria for degradation [73,74]. PINK1 accumulation on dysfunctional mitochondria initiates a protective cascade by phosphorylating and subsequently activating Parkin, an E3 ubiquitin ligase. This activation results in the tagging of mitochondrial surface proteins with ubiquitin, effectively marking the damaged mitochondria for degradation through the lysosomal pathway. This precise clearance mechanism is essential for mitochondrial quality control. However, in Parkinson’s disease (PD), disruptions in PINK1/Parkin signalling lead to a failure in removing dysfunctional mitochondria, contributing to cellular stress and neurodegeneration [75]. Adding to this, research has implicated HMGB1 as a significant upstream regulator of mitophagy, acting through pathways that intersect with PINK1/Parkin function. In zebrafish models exposed to the mitochondrial toxin MPTP, HMGB1 co-activated mitophagy and neuroinflammatory responses through the HMGB1/TLR-4/NF-κB pathway, leading to an upregulation of both PINK1 and Parkin, suggesting a direct influence on the mitophagy machinery [76]. Moreover, as previously mentioned, HMGB1 has been shown to influence mitochondrial morphology and bioenergetics via interaction with HSPβ1 [27,77].

Interestingly, when conceptualized as a mtDAMP, HMGB1 exhibits a dual role in mitochondrial quality control. It can be both released by stressed mitochondria and act as a signalling molecule that helps regulate mitophagy [78].

**Figure 2.**
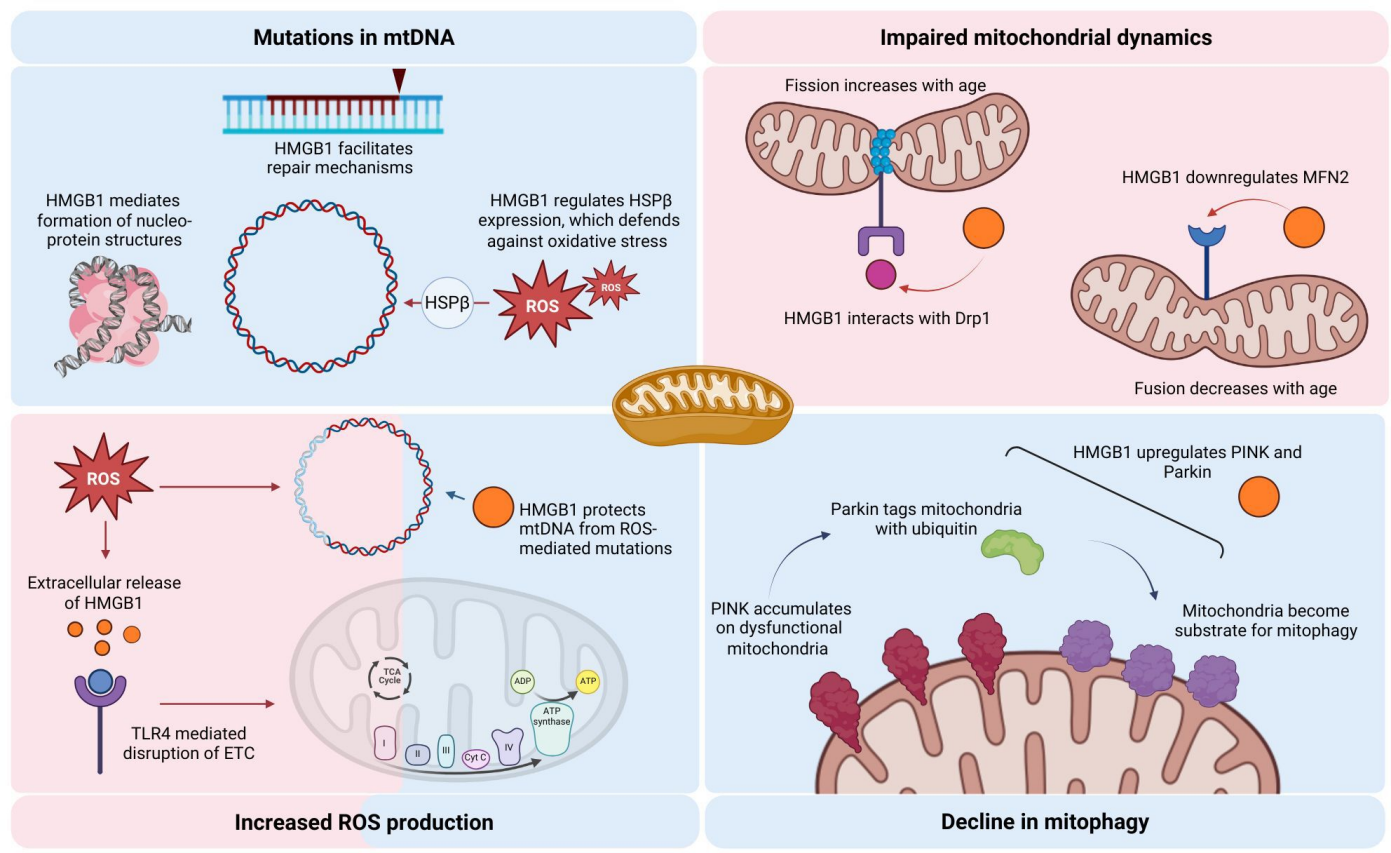
Association of HMGB1 with factors contributing to mitochondrial dysfunction. The figure shows beneficial (blue) and detrimental (red) roles of HMGB1 in various mechanisms underlying age-related mitochondrial dysfunction. Figure created using BioRender.

The dual role of HMGB1 in the context of age-related mitochondrial dysfunction is summarized in figure 2.

### 2.6. Cross-talk between mitochondrial dysfunction, HMGB1 and the hallmarks of ageing

HMGB1 may play a pivotal role in facilitating the cross-talk between mitochondrial dysfunction and other hallmarks of ageing, potentially amplifying the interconnected deterioration seen in ageing cells. We have already discussed the link between mitochondrial dysfunction, HMGB1 and genomic instability as well as chronic inflammation.

The elevated ROS production arising from mitochondrial dysfunction accelerates telomere shortening and damage, contributing to cellular senescence [79]. Meanwhile, HMGB1 plays a role in maintaining telomere integrity, as evidenced by HMGB1-deficient cells exhibiting reduced telomerase activity and telomere shortening [80]. However, whereas this suggests that HMGB1 plays a role in preserving telomere function and chromosomal stability, the lack of telomeres in mtDNA dispels this idea in the mitochondrial context. However, increased cellular ROS levels impact DNA methylation patterns, driving age-associated epigenetic modifications [81,82]. Although current literature does not directly link HMGB1 to these alterations, the association between elevated ROS and epigenetic shifts in both the nuclear and mitochondrial genomes, and the ROS-induced HMGB1 release and damage previously discussed in this review, raises the possibility that HMGB1 may play a role in mediating or responding to these age-related changes, especially while it functions as a DNA chaperone.

Nutrient sensing is regulated by the mammalian target of rapamycin (mTOR) and is intricately connected to mitochondrial function [83]. mTOR is central to this pathway, regulating mitochondrial activity, biogenesis and dynamics by promoting the synthesis of key mitochondrial proteins, including mitochondrial transcription factor A (TFAM), ribosomal proteins and components of complexes I and V [84]. Excessive activity of the mTOR pathway over time contributes to mitochondrial dysfunction via increased TFAM production. Structurally similar to HMGB1, TFAM synergizes with mtDNA released from dysfunctional mitochondria to activate immune cells via TLR-9 and RAGE—both receptors central to inflammatory signalling [21,85]. This parallel extends to HMGB1, which also interacts with TLR-9 and RAGE following binding to mtDNA, triggering inflammatory responses [50]. Furthermore, HMGB1 also directly stimulates the PI3K/AKT/mTOR pathway [86]. Therefore, through both direct and receptor-mediated pathways, HMGB1 appears to link nutrient-sensing disruptions to mitochondrial dysfunction and immune activation, demonstrating its multifaceted impact on the ageing landscape.

Mitochondrial dysfunction-induced cellular senescence, driven by various factors, is collectively referred to as mitochondrial dysfunction-associated senescence (MiDAS) [87]. Unlike the typical senescence-associated secretory phenotype (SASP), MiDAS cells exhibit reduced NAD+/NADH ratios, which contribute to cell growth arrest, and lack the IL−1-dependent inflammatory arm of SASP [88]. However, dysfunctional mitochondria link cellular senescence to inflammation through the release of DAMPs [89]. mtDNA is released from damaged mitochondria where it can activate key inflammatory pathways, such as both the NLRP3 inflammasome and the cGAS/STING pathway, resulting in the secretion of pro-inflammatory cytokines and the propagation of SASP [90–92]. With recognition of HMGB1 as an mtDAMP, it may be associated with mitochondrial dysfunction-induced cellular senescence. HMGB1 is known to lead to excessive ROS production that activates TLR-4 and RAGE receptor pathways, subsequently inducing NLRP3 inflammasome activation [93]. Moreover, HMGB1 has been shown to activate the STING pathway, which drives senescence through STAT6 and p21 [94]. These processes highlight the potential role of HMGB1 in linking mitochondrial dysfunction to the inflammatory and growth-arrest features of cellular senescence.

Stem cell exhaustion, like other hallmarks of ageing, is intricately connected to elevated ROS production [95]. Interestingly, while HMGB1 is closely associated with excessive ROS levels, recent research highlights a potentially restorative role for its Box A domain. Overexpression of this domain in mesenchymal cell models has been shown to upregulate key stemness markers, including OCT4, NANOG and SOX2, while also enhancing cellular proliferation [96]. These findings suggest that HMGB1, particularly through its Box A domain, may play a pivotal role in preserving stem cell function and mitigating stem cell exhaustion.

As our understanding of ageing grows, it becomes increasingly clear that the interplay between mitochondrial dysfunction, HMGB1, and the hallmarks of ageing extends beyond individual cells to influence systemic processes. Among these, extracellular vesicle (EV) secretion is being considered as next-generation candidate to broaden the classic hallmarks of ageing [97]. EVs play a pivotal role in cell-to-cell communication, particularly in propagating stress and inflammatory signals across tissues—a process that can be influenced by mitochondrial dysfunction and the associated rise in ROS levels. Interestingly, HMGB1 has been identified within EVs, where it acts as a mediator of intercellular signalling in both inflammatory and senescent environments. The release of HMGB1 through EVs, as shown in studies on acute liver injury and cortical stress [98,99], reveals a pathway through which mitochondrial dysfunction and HMGB1 may extend their influence beyond individual cells, potentially contributing to age-related cellular dysfunctions on a systemic level.

**Figure 3.**
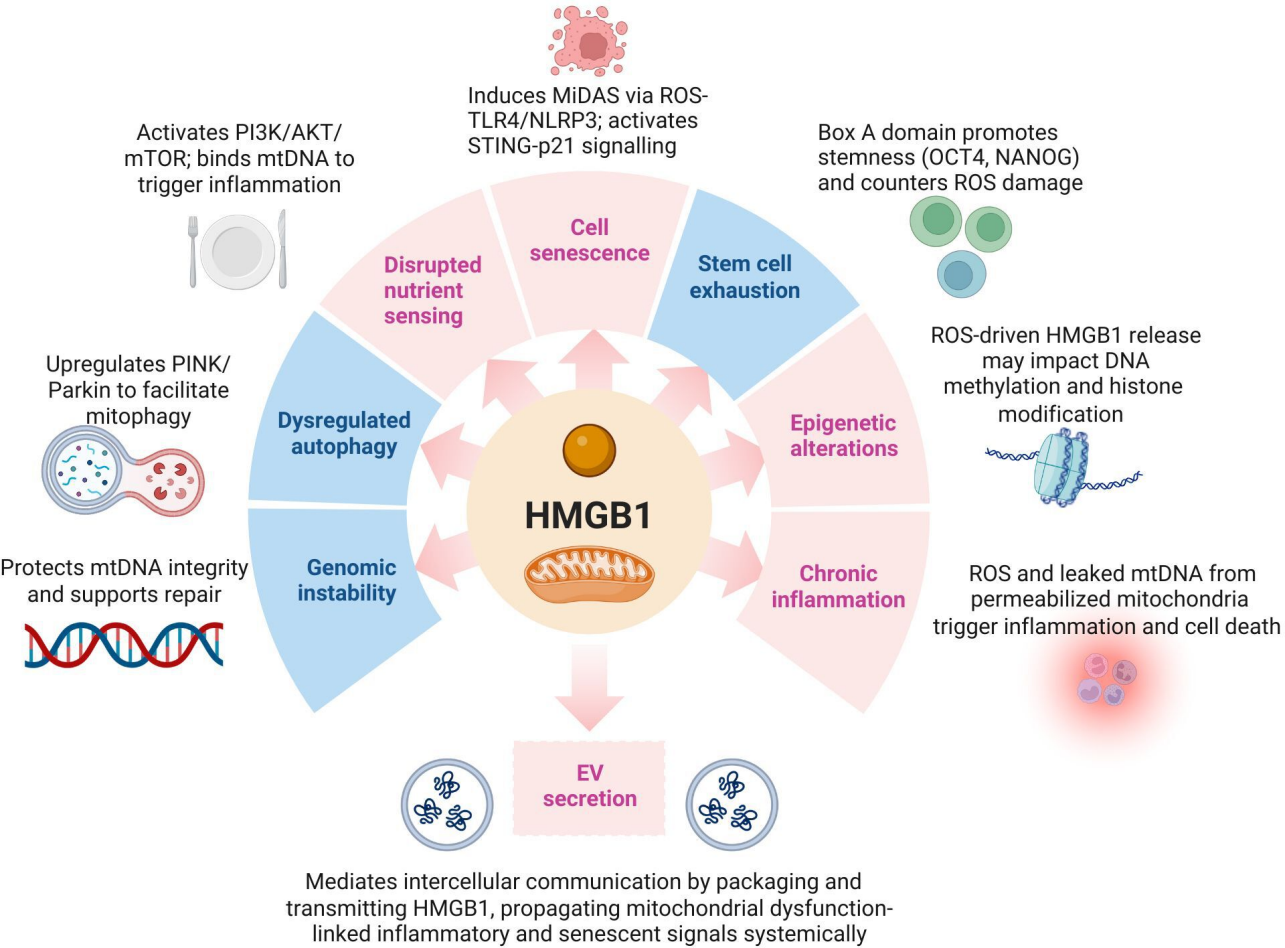
HMGB1 as a central mediator linking mitochondrial dysfunction to the hallmarks of ageing. The figure summarizes the role of HMGB1 in connecting mitochondrial dysfunction to various hallmarks of ageing. This underscores its multifaceted role in protecting against (blue) and exacerbating (red) age-related cellular and systemic changes. Figure created using BioRender.

It can be seen that HMGB1 has a multifaceted role in linking mitochondrial dysfunction to key hallmarks of ageing (figure 3). It can both exacerbate and mitigate age-related cellular changes, depending on the cellular context. Differences in HMGB1 oxidation state may partly underlie this duality, with the fully reduced isoform favouring reparative and homeostatic functions, and the disulfide isoform driving pro-inflammatory responses. Moreover, multiple PTMs regulate its nuclear-cytoplasmic localization and secretion, enabling context-dependent intracellular or extracellular roles. HMGB1 functions to protect cellular integrity by supporting mtDNA repair, facilitating mitophagy via the PINK1/Parkin pathway, and preserving stem cell function through its Box A domain. These actions help maintain cellular homeostasis and mitigate the impact of mitochondrial dysfunction. However, HMGB1 also contributes to the progression of age-related dysfunctions by promoting chronic inflammation, enhancing cellular senescence via the NLRP3 inflammasome and STING pathway, and potentially influencing epigenetic alterations driven by ROS-induced damage. Additionally, the presence of HMGB1 in EVs facilitates the propagation of mitochondrial dysfunction-associated inflammatory signals to other cells, extending the effects of age-related cellular damage at the organ level.

In conclusion, HMGB1 plays a pivotal role in mitochondrial health, acting as both a protector and a contributor to dysfunction. Its roles in safeguarding mtDNA integrity and regulating mitochondrial bioenergetics and dynamics underscore its significance in maintaining cellular health. However, its shift to a pro-inflammatory mtDAMP under oxidative stress highlights a dual nature. Beyond mitochondrial dysfunction, HMGB1 emerges as a central mediator linking this dysfunction to other hallmarks of ageing. This multifaceted role positions HMGB1 as both a marker and a modulator of age-related decline, making it a compelling target for therapeutic strategies. Harnessing protective functions of HMGB1 while mitigating its pathological effects holds promise for strategies aimed at restoring mitochondrial function, reducing systemic inflammation and promoting resilience against age-related diseases. Future research unravelling these mechanisms may open transformative avenues for interventions to extend healthspan and improve quality of life in ageing populations.

## Data Availability

Supplementary material is available online [100].
